# Clinical Outcomes of Palliative Radiotherapy for Breast Lesions in Symptomatic Advanced Breast Cancer: A Decade of Experience at a Regional Tertiary Hospital

**DOI:** 10.3390/cancers18050769

**Published:** 2026-02-27

**Authors:** Yoon Young Jo, Jae Won Park, Ji Woon Yea, Se An Oh, Jaehyeon Park

**Affiliations:** Department of Radiation Oncology, Yeungnam University Medical Center, Yeungnam University College of Medicine, Daegu 42415, Republic of Korea

**Keywords:** breast neoplasms, palliation, radiotherapy, quality of life, survival

## Abstract

**Background/Objectives:** Palliative radiotherapy (RT) to the breast or chest wall is an effective option for symptom relief in advanced or metastatic breast cancer. However, real-world data on clinical outcomes, prognostic factors, and optimal RT dose and timing in the modern systemic therapy era remain limited. This study aimed to evaluate the efficacy of palliative breast RT and explore the optimal integration of systemic therapy with RT. **Methods:** We retrospectively reviewed 38 patients treated with palliative RT between 2015 and 2024 at a regional tertiary center. The median gross tumor volume (GTV) dose was 50 Gy (range, 30–62.5 Gy), corresponding to a median biologically effective dose (BED) to the GTV (α/β = 4) of 78.9 Gy. Treatment response, symptom relief, toxicity, and survival outcomes were analyzed. **Results:** With a median follow-up of 9.5 months, median overall survival (OS) was 12.8 months. 1- and 2-year in-field local control (LC) rates were both 79.6%. In a parsimonious multivariate analysis, ≥3 prior lines of systemic therapy (HR 3.500, 95% CI 1.278–9.590, *p* = 0.015) was independently associated with worse OS, whereas use of SIB or GTV boost was associated with improved OS (HR 0.351, 95% CI 0.145–0.848, *p* = 0.020). Higher planning target volume (PTV)-BED (α/β = 4) correlated with improved LC (HR 0.909, 95% CI 0.839–0.985, *p* = 0.019). Symptom relief within 3 months occurred in 82%. Treatment-related toxicities were generally mild, with no grade ≥ 4 acute or grade ≥ 2 late toxicities observed. **Conclusions:** Palliative breast RT achieved durable local control and effective symptom palliation with acceptable toxicity. Dose-tailored RT and earlier integration within the disease course may optimize outcomes in selected patients.

## Simple Summary

Patients with advanced breast cancer often experience severe local symptoms, such as pain, bleeding, ulceration, and malodorous discharge, that significantly reduce quality of life. Palliative radiotherapy is used to control these symptoms, but its optimal dose and timing remain unclear. In this real-world study, radiotherapy provided effective local control with acceptable toxicity, and higher radiation doses with boost techniques were associated with improved local outcomes. Patients who had received multiple lines of systemic therapy before radiotherapy had worse overall survival, suggesting limited benefit when treatment is delayed. These findings highlight the importance of integrating palliative radiotherapy at an appropriate time in the disease course.

## 1. Introduction

Breast cancer is the most commonly diagnosed malignancy among women worldwide, with approximately 2.3 million new cases and over 660,000 deaths reported globally in 2022 [1]. Breast cancer development is driven by complex molecular and inflammatory processes within the tumor microenvironment, including cytokine-mediated signaling pathways that promote tumor initiation and progression. Despite advances in screening and early detection, approximately 10–30% of patients present with locally advanced breast cancer. Within this subset, a clinically significant portion of patients present with de novo stage IV disease [2,3]. Patients with advanced breast cancer frequently experience distressing symptoms, such as fungating or ulcerating masses, malodorous discharge, pain, and bleeding, which markedly impair quality of life (QOL). In addition, these symptoms cause significant psychological distress, including depression, shame, and social isolation [4]. Palliative radiotherapy (RT) is a well-established and effective modality for alleviating such symptoms and achieving local disease control in advanced breast cancer [5,6,7]. However, unlike palliative RT for bone or brain metastases, there is currently no standardized consensus or breast-specific guidelines.

Over the past decade, the therapeutic landscape of metastatic breast cancer has evolved substantially. The introduction of modern systemic agents, including antibody–drug conjugates such as trastuzumab-deruxtecan and CDK4/6 inhibitors, has led to significant improvements in progression-free survival (PFS) and overall survival (OS) for patients with stage IV disease [8,9]. As survival durations increase, the clinical importance of achieving durable local control of the primary breast lesion has become more pronounced. Consequently, temporary symptom relief may no longer be sufficient; instead, long-term local control is increasingly required to prevent the recurrence of distressing symptoms over an extended survival period and to maintain sustained QOL. In this context, durable local control refers not merely to short-term tumor regression, but to sustained stabilization of the primary breast lesion sufficient to prevent recurrent bleeding, infection, or ulceration, and to allow uninterrupted continuation of systemic therapy.

However, clinical practice has not yet fully adapted to this shifting paradigm, leaving several critical questions unanswered. First, the optimal timing of palliative RT in the disease trajectory—whether it should be implemented earlier during systemic therapy or deferred until after failure of multiple lines of treatment—has not been adequately investigated [10]. In contemporary practice, palliative breast RT is often reserved as a late palliative option, particularly in heavily pretreated patients, yet this strategy may limit its potential benefits. Second, although the primary intent of RT in this setting remains symptom palliation, there is ongoing debate over whether achieving robust and durable local control of the primary tumor can translate into an OS benefit. Several studies, including both retrospective analyses and a randomized controlled trial, have suggested that aggressive local treatment may improve OS by reducing overall tumor burden or preventing life-threatening local complications [11,12,13,14]. However, other randomized controlled trials have failed to demonstrate a significant survival advantage, resulting in ongoing controversy [15,16].

Therefore, in this study, we aimed to evaluate the clinical outcomes of tailored palliative RT for patients with symptomatic advanced breast cancer using a decade-long, real-world dataset from a regional tertiary hospital.

## 2. Materials and Methods

### 2.1. Patients

This study was a retrospective, single-institution analysis conducted at a regional tertiary hospital. Patients with symptomatic advanced breast cancer treated with palliative RT between January 2015 and December 2024 were included. The present study protocol was reviewed and approved by the Institutional Review Board of our medical center (approval number: YUMC 2025-10-041). Given the study’s retrospective design, the need for informed consent was waived. All eligible patients treated for symptomatic locoregional disease during this period were screened for inclusion. Patients were eligible if they had symptomatic breast, chest wall, and/or regional nodal disease requiring palliative RT. Symptoms prompting treatment included pain, tissue hardness, ulceration or fungating masses, bleeding, malodorous discharge, or limitation in the range of arm motion. Patients were excluded if they had no locoregional symptoms, if radiotherapy was delivered exclusively to distant metastatic sites without breast or chest wall involvement, or if treatment was administered with curative intent.

### 2.2. Radiation Therapy

All patients underwent computed tomography (CT) simulation in the supine position, with immobilization devices to ensure a reproducible setup and patient comfort. The gross tumor volume (GTV) was delineated based on clinical examination and available imaging studies. The clinical target volume (CTV) encompassed the GTV with or without inclusion of adjacent regional nodal areas at risk and elective areas at high risk for subclinical spread. The planning target volume (PTV) encompassed the CTV with appropriate margins. RT was delivered primarily using three-dimensional conformal radiotherapy (3D-CRT) or intensity-modulated radiotherapy (IMRT). Dose–fractionation schedules were individualized based on performance status, disease burden, symptom severity, prior treatments, and anticipated prognosis. Simultaneous integrated boost (SIB) or sequential GTV boost techniques were employed when deemed clinically appropriate. To allow comparisons of dose effects of various fractionation schedules, the BED was calculated using an α/β ratio of 4 Gy for tumor control. Cumulative BED values were calculated separately for the GTV and PTV to allow comparison across heterogeneous fractionation schedules.

### 2.3. Outcome Definitions

We evaluated OS, local control (LC), PFS, and distant metastasis-free survival (DMFS) rates. OS was defined as the time from the first day of RT to death from any cause. LC was defined as the time from the first day of RT to disease progression in the irradiated field. PFS was defined as the time from the first day of RT to any disease progression. DMFS was defined as the time from the first day of RT to the development of new distant metastatic disease. Objective radiologic response was assessed within 6 months after completion of RT using imaging studies. For patients with multiple post-treatment imaging assessments, the best response was recorded. Radiologic response was evaluated according to Response Evaluation Criteria in Solid Tumors (RECIST), version 1.1 [17], based on changes in lesion size compared with baseline pre-RT imaging. Symptom relief was defined based on electronic medical records as overall improvement in presenting symptoms within 3 months after RT. This variable was analyzed as a binary endpoint. Treatment-related toxicities were graded according to the Common Terminology Criteria for Adverse Events, version 5.0 [18]. Acute toxicity was defined as events occurring within 3 months of the start of RT, whereas late toxicity was defined as events occurring after 3 months.

### 2.4. Statistical Analyses

Survival outcomes, including OS, LC, PFS, and DMFS rates, were analyzed using the Kaplan–Meier method and compared using the log-rank test. For Kaplan–Meier analyses, OS was compared between patients receiving a GTV-BED of ≥80 Gy and those receiving <80 Gy. Similarly, LC was compared between patients receiving a GTV-BED of ≥65 Gy versus < 65 Gy, and a PTV-BED of ≥70 Gy versus < 70 Gy. Dose cut-off values for Kaplan–Meier analyses were determined using receiver operating characteristic (ROC) curve analysis, with optimal thresholds selected based on the Youden index. Previously reported dose ranges in the literature were also considered to ensure clinical interpretability. To identify predictors of survival outcomes, univariate and multivariate analyses were performed using Cox proportional hazards models, and estimated hazard ratios (HRs) with 95% confidence intervals (CIs) were reported. For multivariate analysis, variables with *p*-values < 0.05 in the univariate analysis were included in the initial model, and a backward elimination method was employed to identify independent predictors. In addition to the exploratory multivariable model including variables significant in univariate analysis, a parsimonious multivariable model restricted to clinically relevant covariates was constructed to evaluate the stability of the estimates and reduce the risk of overfitting. All statistical analyses were performed using IBM SPSS Statistics for Windows, version 29.0 (IBM Corp., Armonk, NY, USA). A two-sided *p*-value < 0.05 was considered statistically significant. Additional sensitivity analyses were conducted using Firth penalized Cox proportional hazards regression implemented in R software, version 4.5.2 (R Foundation for Statistical Computing, Vienna, Austria) using the ‘coxphf’ package.

## 3. Results

### 3.1. Patient, Tumor, and Treatment Characteristics

Between 2015 and 2024, 38 patients with symptomatic advanced breast cancer were treated with palliative RT. Baseline characteristics are summarized in Table 1. At initial diagnosis, most patients presented with high-burden disease, including T4 (65.8%) and N3 (73.7%) stages. While nine patients had M0 disease at diagnosis, the majority presented with de novo M1 disease. The median interval from initial diagnosis to RT initiation was 14.3 months. Patients were heavily pretreated, with 50% having received more than two lines of systemic therapy before RT. At the time of RT initiation, all patients had symptomatic local disease. Pain and tissue hardness were universal, and many also exhibited ulcerating or fungating lesions, discharge, or bleeding, indicating significant locoregional tumor burden. RT target volumes most commonly included the breast or chest wall with regional nodes. Various fractionation schedules were individualized according to clinical circumstances. The median cumulative BED (α/β = 4) was 78.9 Gy for the GTV and 75.0 Gy for the PTV. Advanced RT techniques were predominantly utilized, treatment compliance was high, and concurrent systemic therapy was administered in approximately half of the patients.

### 3.2. Survival Outcome and Local Control

**Figure 1:**
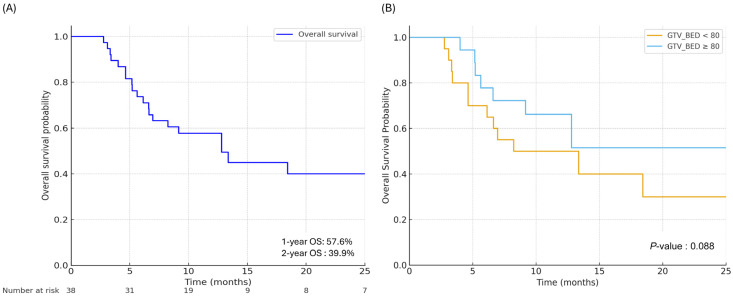
Overall survival. (**A**) Overall survival of the entire patient cohort. (**B**) Overall survival stratified by gross tumor volume biologically effective dose (GTV-BED), comparing patients treated with GTV-BED of <80 Gy versus ≥80 Gy. OS, overall survival; GTV, gross tumor volume; BED, biologically effective dose; LC, local control.

The median OS for the entire cohort was 12.8 months, with 1- and 2-year OS rates of 57.6% and 39.9%, respectively (Figure 1A). When stratified by a GTV-BED cutoff of 80 Gy, the high-dose group (≥80 Gy) showed superior OS compared to the low-dose group (<80 Gy), although this difference did not reach statistical significance (*p* = 0.088, Figure 1B). In the univariate analysis, worse OS was significantly associated with an Eastern Cooperative Oncology Group (ECOG) performance status of >2 (HR 3.027, 95% CI 1.165–7.867, *p* = 0.023), ER-negative/HER2-negative subtype (HR 3.954, 95% CI 1.729–9.045, *p* = 0.001), receipt of ≥3 lines of systemic therapy prior to radiotherapy (HR 2.687, 95% CI 1.020–7.082, *p* = 0.046), and the presence of ≥3 presenting symptoms at the time of radiotherapy (HR 2.332, 95% CI 1.038–5.240, *p* = 0.040). Conversely, the use of SIB or GTV boost (HR 0.415, 95% CI 0.175–0.988, *p* = 0.047) and early symptom relief (HR 0.203, 95% CI 0.078–0.527, *p* = 0.001) were associated with improved OS. In the multivariate analysis, ≥3 prior lines of systemic therapy, ≥3 presenting symptoms, post-RT early symptom relief, and use of SIB or GTV boost were significantly associated with OS. To mitigate potential overfitting, a parsimonious multivariable model was used for OS. In this model, receipt of ≥3 prior lines of systemic therapy before RT was associated with inferior OS (HR 3.500, 95% CI 1.278–9.590, *p* = 0.015), whereas use of SIB or GTV boost was associated with improved OS (HR 0.351, 95% CI 0.145–0.848, *p* = 0.020). Sensitivity analysis using Firth-penalized Cox regression yielded consistent results, with ≥3 prior lines of systemic therapy remaining significantly associated with worse OS (HR 3.632, 95% CI 1.285–9.387, *p* = 0.017), while use of SIB or GTV boost remained associated with improved OS (HR 0.363, 95% CI 0.146–0.837, *p* = 0.017). Detailed results are shown in Supplementary Table S1.

**Figure 2:**
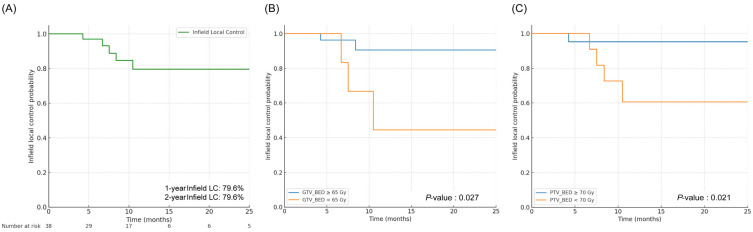
In-field local control. (**A**) In-field local control of the entire patient cohort. (**B**) In-field local control stratified by gross tumor volume biologically effective dose (GTV-BED), comparing patients treated with GTV-BED of <65 Gy versus ≥65 Gy. (**C**) In-field local control stratified by planning target volume biologically effective dose (PTV-BED), comparing patients treated with PTV-BED of <70 Gy versus ≥ 70 Gy. LC, local control; GTV, gross tumor volume; BED, biologically effective dose.

The estimated 1-year and 2-year infield LC rates were both 79.6%, indicating durable local disease control following palliative RT (Figure 2A). Stratification by GTV dose showed that patients receiving a GTV-BED of ≥65 Gy demonstrated significantly superior LC compared with those receiving <65 Gy (*p* = 0.027; Figure 2B). Similarly, stratification by PTV dose revealed that patients receiving a PTV-BED of ≥70 Gy had significantly improved local control compared with those receiving <70 Gy (*p* = 0.021; Figure 2C). Result of the univariate and multivariate analyses of LC are summarized in Supplementary Table S2. In univariate analysis, most baseline clinicopathologic factors, including age, ECOG status, tumor stage at RT, metastatic burden, and RT target volume, were not significantly associated with LC. However, higher cumulative doses to the PTV were significantly associated with improved LC (HR 0.872 per Gy, 95% CI 0.772–0.984, *p* = 0.026). Similarly, higher cumulative BED to the PTV was associated with superior LC (HR 0.909, 95% CI 0.839–0.985, *p* = 0.019). In addition, symptom relief within 3 months after palliative radiotherapy was associated with a significantly lower risk of local failure (HR 0.108, 95% CI 0.017–0.667, *p* = 0.017). In multivariate analysis, cumulative BED to the PTV remained an independent predictor of improved LC (HR 0.909, 95% CI 0.839–0.985, *p* = 0.019), while other clinical and treatment-related variables did not reach statistical significance.

**Figure 3:**
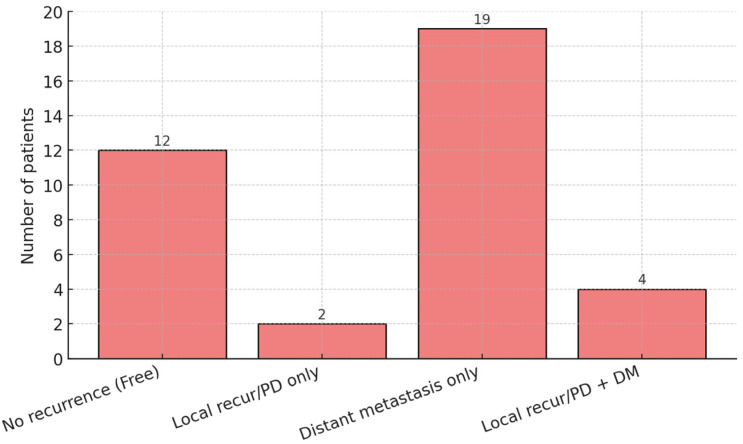
Distribution of first recurrence sites. Data represent absolute number of patients. PD, progressive disease; DM, distant metastasis.

The median PFS was 7.5 months in the entire population, with estimated 1-year and 2-year PFS rates of 34.2% and 18.0%, respectively (Supplementary Figure S1). The distribution of first disease progression sites is summarized in Figure 3. Among 37 evaluable patients, 19 (51.4%) experienced distant metastases only as the first site of progression, whereas 2 (5.4%) developed isolated local recurrence or local progression. Combined local progression and distant metastases occurred in 4 patients (10.8%), and 12 patients (32.4%) remained progression-free during follow-up. In univariate analysis, longer time from initial diagnosis was associated with improved PFS (HR 0.968 per month, 95% CI 0.939–0.999, *p* = 0.041), while the ER-negative/HER2-negative subtype was associated with significantly worse PFS (HR 1.410, 95% CI 1.156–1.719, *p* < 0.001). Patients receiving concurrent systemic therapy during RT demonstrated significantly improved PFS compared with those who did not (HR 0.345, 95% CI 0.161–0.739, *p* = 0.006). In addition, a change in systemic therapy regimen at the initiation of RT was associated with inferior PFS (HR 4.314, 95% CI 1.248–14.913, *p* = 0.021), and early symptom relief within 3 months after RT was associated with improved PFS (HR 0.323, 95% CI 0.133–0.786, *p* = 0.013). In the multivariate analysis, longer time from initial diagnosis remained independently associated with improved PFS (HR 0.871, 95% CI 0.764–0.992, *p* = 0.037), while the ER-negative/HER2-negative subtype was an independent predictor of worse PFS (HR 42.483, 95% CI 3.271–551.716, *p* = 0.004). Other variables did not retain statistical significance in the adjusted model (Supplementary Table S3).

DMFS was also assessed (Supplementary Figure S2). The median DMFS was 8.2 months, with estimated 1-year and 2-year DMFS rates of 41.0% and 25.6%, respectively. In univariate analysis, the ER-negative/HER2-negative subtype was strongly associated with inferior DMFS (HR 5.139, 95% CI 2.096–12.596, *p* < 0.001). Patients who had received ≥2 lines of systemic therapy prior to RT demonstrated worse DMFS than those receiving fewer lines (HR 3.354, 95% CI 1.363–8.254, *p* = 0.008). In addition, switching the systemic therapy regimen at the initiation of RT was associated with significantly worse DMFS (HR 6.010, 95% CI 1.588–22.750, *p* = 0.008). Conversely, concurrent systemic therapy during RT was associated with improved DMFS (HR 0.425, 95% CI 0.188–0.961, *p* = 0.040). Early symptom relief after RT was also associated with improved DMFS (HR 0.293, 95% CI 0.112–0.766, *p* = 0.012). In the multivariate analysis, the ER-negative/HER2-negative subtype (HR 20.941, 95% CI 3.232–135.696, *p* = 0.001) and a change in systemic therapy during RT (HR 7.191, 95% CI 1.304–39.638, *p* = 0.024) remained independently associated with inferior DMFS (Supplementary Table S4).

### 3.3. Objective Treatment Response Assessments and Treatment-Related Toxicities

Objective treatment response was assessed based on radiologic evaluation performed within 6 months after completion of palliative RT. Among 35 evaluable lesions, an objective radiologic response was observed in 18 lesions (51%), including 2 complete responses and 16 partial responses. Stable disease was observed in 15 lesions (43%), whereas progressive disease occurred in 1 lesion (3%), and mixed response was observed in 1 lesion (3%). Overall, most treated lesions demonstrated either tumor regression or disease stabilization following palliative RT, indicating meaningful local disease control despite the advanced disease status of patients. When radiologic response was further examined according to the prescribed radiotherapy regimen, complete responses were observed in lesions treated with 50 Gy in 25 fractions, followed by a 10 Gy boost, and with 56/46 Gy in 20 fractions. Detailed response patterns according to treatment regimen are summarized in Supplementary Table S5. Treatment-related toxicities were generally mild and manageable (Supplementary Table S6). Acute toxicities were predominantly low grade, with 26 patients (68.4%) experiencing no acute toxicity. Grade 1 acute toxicity was observed in 2 patients (5.3%), and grade 2–3 acute toxicity occurred in 10 patients (26.3%). No grade ≥ 4 acute toxicity was observed. With respect to late toxicities, 30 patients (78.9%) experienced no late toxicity, whereas grade 1 late toxicity was observed in 8 patients (21.1%). Importantly, no grade ≥ 2 late toxicities were observed during follow-up. Overall, palliative radiotherapy was well tolerated, with a low incidence of clinically significant acute or late adverse events.

## 4. Discussion

In this retrospective analysis of symptomatic advanced breast cancer, we demonstrated that tailored palliative RT achieved meaningful symptom relief and durable LC in a real-world dataset treated in the modern systemic therapy era. Importantly, our findings suggest that RT dose intensity, particularly when assessed using BED-based metrics and boost strategies, was significantly associated with improved local control and was favorable in terms of OS. In addition, early symptom relief following RT emerged as a strong prognostic marker for multiple oncologic outcomes. Collectively, these results support a more proactive and individualized role for palliative breast RT beyond its traditional use as a late salvage modality.

One of the most notable findings of this study was the clear association between RT dose intensity and in-field LC. Patients receiving a GTV-BED of ≥65 Gy or a PTV-BED of ≥70 Gy experienced significantly superior LC compared with those treated with lower BED regimens. Furthermore, cumulative BEDs to the PTV remained independent predictors of LC on multivariate analysis. These findings support the existence of a dose–response relationship even in the palliative setting, challenging the conventional paradigm that short-course, low-dose RT is universally sufficient for symptom management. Previous studies of palliative breast RT have reported favorable local responses but have been limited by heterogeneous fractionation schemes and a lack of quantitative dose–response analyses. Hoeltgen et al. [10] summarized institutional experiences demonstrating symptom improvement and acceptable toxicity, but did not identify optimal dose thresholds for durable LC. Similarly, Jacomina et al. [6] reported effective palliation with various regimens but emphasized the absence of standardized dose recommendations. By applying BED-based comparisons across diverse fractionation schedules, our study provides clinically relevant evidence that dose escalation, when feasible, may improve long-term local outcomes in symptomatic advanced breast cancer.

Early symptom relief within three months after RT was strongly associated with improved OS, LC, PFS, and DMFS in our patients. This observation suggests that symptom response is not merely a subjective endpoint but may reflect underlying tumor radiosensitivity, adequate dose delivery, and favorable disease biology. Similar associations between symptomatic response and survival outcomes have been reported in other palliative RT settings, including bone and thoracic malignancies, where early clinical improvement has been correlated with longer survival [19,20,21]. In the context of advanced breast cancer, early symptom relief may serve as a pragmatic and readily assessable surrogate marker for treatment effectiveness. This has important clinical implications, as symptom response could help guide subsequent management decisions, including the continuation or modification of systemic therapy and the intensity of follow-up. Our findings support incorporating early symptom assessment as a meaningful outcome measure in future palliative breast RT studies.

Another key contribution of this study is its exploration of the timing of palliative RT throughout the disease course. Patients who had received three or more lines of systemic therapy prior to RT demonstrated significantly inferior OS and DMFS, whereas the use of concurrent systemic therapy during RT was associated with improved PFS and DMFS. In contrast, changes in systemic therapy during RT were associated with worse outcomes, likely reflecting aggressive disease biology or rapid progression. These findings suggest that palliative RT may confer greater benefit when integrated earlier in the disease trajectory rather than being reserved as a late palliative intervention. In contemporary practice, palliative breast RT is often deferred until multiple systemic therapies have failed, at which point patients may have poor performance status and limited life expectancy. Our data support reconsidering this approach and suggest that earlier integration of RT, particularly in patients with symptomatic primary tumors and ongoing systemic treatment, may maximize both local and systemic disease control. Despite the widespread use of palliative RT in metastatic breast cancer, few studies have addressed optimal treatment timing. Most existing literature focuses on symptom outcomes without contextualizing RT within the broader systemic therapy sequence [10,22]. Our results provide real-world evidence to inform this clinical decision-making gap.

Although palliative RT is primarily intended for symptom relief, our study demonstrated that RT-related factors, including the use of SIB or GTV boost, were independently associated with improved OS. However, given the retrospective design, treatment intensification may have been preferentially offered to patients with more favorable clinical characteristics, such as better performance status or lower systemic disease burden. Therefore, the association between boost strategies and improved survival should be interpreted cautiously in the context of potential residual confounding. Nonetheless, these findings raise the hypothesis that durable local control of the primary tumor may contribute to survival benefits in selected patients. Potential mechanisms include reduction of overall tumor burden, prevention of local complications such as infection or bleeding, and improved ability to maintain systemic therapy without interruption due to uncontrolled local symptoms or hospitalization. Similar hypotheses have been proposed for other malignancies, where aggressive local treatment of the primary tumor in oligometastatic settings has been associated with improved survival outcomes [23]. In the era of prolonged survival enabled by modern systemic therapies, maintaining local disease stability may play an increasingly important supportive role in comprehensive cancer care.

Certain limitations of this study should be acknowledged. First, the retrospective and single-institution design introduced the possibility of selection bias. Second, the relatively small sample size reflects the specific clinical scenario of symptomatic advanced breast cancer requiring palliative RT and may limit the generalizability of our findings. Third, symptom relief was assessed based on medical record documentation, which is inherently subject to reporting variability and potential underestimation of symptom burden. Finally, although BED-based analyses provide valuable insights into dose–response relationships, the optimal dose thresholds identified in this study require validation in larger, prospective cohorts. Although we adopted a parsimonious modeling strategy to mitigate overfitting, the relatively small sample size and limited number of events remain important limitations of this study. However, additional sensitivity analyses demonstrated consistent findings, supporting the robustness of the observed associations. Therefore, these results should be interpreted with appropriate caution and validated in larger prospective cohorts.

Future research should focus on identifying the optimal timing of local radiotherapy in patients receiving systemic therapy. In particular, it remains unclear at which point during systemic treatment local dose escalation may provide maximal clinical benefit. Prospective studies are warranted to determine whether earlier intervention—before substantial tumor progression, skin invasion, or ulceration—could improve local control and potentially translate into survival benefit. Clarifying patient selection criteria and tumor burden thresholds for initiating dose-intensified palliative radiotherapy will be critical for optimizing treatment strategies in this population.

## 5. Conclusions

In the modern systemic therapy era, palliative RT for symptomatic advanced breast cancer should be reconsidered beyond short-term symptom relief. Our real-world data demonstrate that tailored, dose-aware palliative RT can achieve effective symptom palliation and durable LC. These findings support individualized treatment strategies and may help inform clinical decision-making in the absence of breast-specific palliative RT guidelines.

## Figures and Tables

**Table 1:** Baseline patients and clinical characteristics, N (%) indicates the number of patients and the corresponding percentage.

Characteristics	N (%)
Age (y)	
Median (range)	56.5 (29–79)
ECOG status	
0–1	31 (81.6)
>2	7 (18.4)
Time from initial diagnosis (month)	
Median (range)	14.3 (0.4–78.2)
Clinical T stage at time of diagnosis	
T1	2 (5.3)
T2	6 (15.8)
T3	5 (13.2)
T4	25 (65.8)
Clinical N stage at time of diagnosis	
N0	1 (2.6)
N1	2 (5.3)
N2	7 (18.4)
N3	28 (73.7)
Clinical M stage at time of diagnosis	
M0	9 (23.7)
M1	29 (76.3)
Receptor status	
ER+/HER2−	16 (42.1)
ER+/HER2+	7 (18.4)
ER−/HER2+	1 (2.6)
ER−/HER2−	13 (34.2)
Unknown	1 (2.6)
Histologic grade	
Low	0 (0)
Intermediate	2 (5.3)
High	11 (28.9)
Unknown	25 (65.8)
Histology	
Invasive carcinoma	36 (94.7)
Invasive lobular carcinoma	1 (2.6)
Mucinous carcinoma	1 (2.6)
Recurrence	
Yes	7 (18.4)
No	31 (81.6)
Lines of systemic therapy before RT	
0	2 (5.3)
1	17 (44.7)
2	12 (31.6)
3	3 (7.9)
≥4	4 (10.5)
Prior upfront surgery	
Yes	6 (15.8)
No	32 (84.2)
Clinical T stage at time of RT	
T0	3 (7.9)
T1	1 (2.6)
T2	6 (15.8)
T3	2 (5.3)
T4	26 (68.4)
Clinical N stage at time of RT	
N0	2 (5.3)
N1	2 (5.3)
N2	6 (15.8)
N3	28 (73.7)
Clinical M stage at time of RT	
M0	3 (7.9)
M1	35 (92.1)
Single site, single meta	3 (8.6)
Single site, oligo meta (≤5 sites)	9 (25.7)
Multi-site, oligo meta (≤5 sites)	10 (28.6)
Systemic meta (>5 sites)	13 (37.1)
Presenting symptoms for radiation therapy	
Pain, hardness	38 (100)
Ulcerating or fungating lesion	27 (71.1)
Discharge/Malodor	12 (31.6)
Bleeding	12 (31.6)
Limitation in range of arm motion/lymphedema/brachial plexopathy	6 (15.8)
Median follow-up (month)	
Median (range)	9.5 (1.0–45.1)
Radiation target volume	
Mass only	8 (21.1)
Breast/chest wall only	4 (10.5)
Breast/chest wall and nodes	26 (68.4)
Nodes only	0 (0)
Prescribed RT regimen	
50 Gy/25fx	6 (15.8)
50 Gy/20fx	4 (10.5)
41.6 Gy/16fx → boost 10 Gy/4fx	4 (10.5)
(50/44 Gy)/20fx	4 (10.5)
45 Gy/15fx	3 (7.9)
others	17 (44.7)
Use of SIB or GTV boost	
No	22 (57.9)
SIB	10 (26.3)
GTV boost	6 (15.8)
Cumulative dose to GTV(PTV_RF/SIB) (Gy)	
Median (range)	50 (30–62.5)
Cumulative dose to PTV (Gy)	
Median (range)	45.5 (30–57.5)
Cumulative BED to GTV (Gy), α/β = 4	
Median (range)	78.9 (51.5–111.3)
Cumulative BED to PTV (Gy), α/β = 4	
Median (range)	75.0 (45.0–98.8)
RT technique	
2D	1 (2.6)
3D	7 (18.4)
IMRT	28 (73.7)
VMAT	2 (5.3)
RT compliance	
Complete the RT	32 (84.2)
Incomplete the RT	6 (15.8)
Concurrent systemic therapy	
Yes	21 (55.3)
Endocrine therapy and CDK4/6 inhibitor	11 (52.4)
Capecitabine	3 (14.3)
Halaven	1 (4.76)
Vinorelbine(Navelbine)	1 (4.76)
Palictaxel	1 (4.76)
Kadcyla (T-DM1)	2 (9.5)
Endocrine therapy and Kadcyla	1 (4.76)
Everolimus/Exemestane	1 (4.76)
No	17 (44.7)
Systemic therapy change at the time of RT	
Yes	4 (19.0)
No	17 (81.0)
Symptom relief after RT (within 3 months after palliative RT)	
Yes	31 (81.6)
No	7 (18.4)
Surgery after RT	
Yes	4 (10.5)
No	34 (89.5)

## Data Availability

Research data are stored in an institutional repository and will be shared upon request to the corresponding author.

## Supplementary Materials

The following supporting information can be downloaded at https://www.mdpi.com/article/10.3390/cancers18050769/s1, Supplementary Table S1: univariate and multivariate analyses for Overall survival; Supplementary Table S2: univariate and multivariate analyses for Local control; Supplementary Table S3: univariate and multivariate analyses for Progression-free survival; Supplementary Table S4: univariate and multivariate analyses for Distant metastasis-free survival; Supplementary Table S5. Radiologic response rate (within 6 months after palliative RT); Supplementary Table S6. Treatment-related toxicities; Supplementary Figure S1: Progression-free survival (PFS). Supplementary Figure S2: Distant metastasis-free survival (DMFS).

## Abbreviations

The following abbreviations are used in this manuscript:

QOL	Quality of life
RT	Radiotherapy
PFS	Progression-free survival
OS	Overall survival
BED	Biologically effective dose
CT	Computed tomography
GTV	Gross tumor volume
CTV	Clinical target volume
PTV	Planning target volume
3D-CRT	3-dimensional conformal radiotherapy
IMRT	Intensity-modulated radiotherapy
SIB	Simultaneous integrated boost
LC	Local control
DMFS	Distant metastasis-free survival
HR	Hazard ratio
CI	Confidence interval
ECOG	Eastern Cooperative Oncology Group
