# Supplementary material for: Clinical Outcomes of Palliative Radiotherapy for Breast Lesions in Symptomatic Advanced Breast Cancer: A Decade of Experience at a Regional Tertiary Hospital
Source: Cancers (Basel). 2026 Feb 27;18(5):769. doi: 10.3390/cancers18050769 (PMC12984870; doi:10.3390/cancers18050769)
Supplement: Supplementary file 1 [file cancers-18-00769-s001.zip › Supplementary Tables.pdf]

Supplementary Table S1. univariate and multivariate analyses for Overall Survival

Variable	N(%)	Unadjusted HR (95% CI)	p-value	Adjusted HR† (95% CI)	p-value	Parsimonious HR‡ (95% CI)	p-value	Firth-penalized HR§ (95% CI)	p-value
**Age (y)**									
**Median (range)**	56.5 (29-79)	0.983(0.937-1.031)	0.485						
**ECOG status**									
**0-1**	31 (81.6)	Ref	0.023		0.563				
**>2**	7 (18.4)	3.027(1.165-7.867)							
**Time from initial diagnosis (month)**									
**Median (range)**	14.3 (0.4-78.2)	0.973(0.940-1.008)	0.126						
**Receptor status**									
**Others**	25 (65.8)	Ref	0.001		0.587				
**ER-/HER2-**	13 (34.2)	3.954(1.729-9.045)							
**Histologic grade**									
**Others**	27 (71.1)	Ref	0.849						
**High**	11 (28.9)	1.094(0.434-2.757)							
**Lines of systemic therapy before RT**									
**<3**	31 (81.6)	Ref	0.046	Ref	0.013	Ref	0.015	Ref	0.017
**≥3**	7 (18.4)	2.687(1.020-7.082)		42.081(2.217-798.745)		3.500(1.278-9.590)		3.632(1.285-9.387)	
**Prior upfront surgery**									
**Yes**	6 (15.8)	0.802(0.287-2.246)	0.675						
**No**	32 (68.4)	Ref							
**Clinical T stage at time of RT**									
**Others**	12 (31.6)	Ref	0.589						
**T4**	26 (68.4)	0.787(0.331-1.876)							
**Clinical N stage at time of RT**									
**Others**	10 (26.3)	Ref	0.250						
**N3**	28 (73.7)	2.061(0.601-7.072)							
**Clinical M stage at time of RT**									
**M0**	3 (7.9)	Ref	0.302						
**M1**	35 (92.1)	2.191(0.494-9.719)							
**metastasis (≤5 sites)**	22 (62.9)	Ref	0.141						
**Systemic metastasis (>5 sites)**	13 (37.1)	1.913(0.806-4.537)							
**Number of presenting symptoms**									
**<3**	24 (63.2)	Ref	0.040	Ref	0.011				
**≥3**	14 (36.8)	2.332(1.038-5.240)		32.705(2.256-474.092)					
**Radiation target volume**									
**Mass only, Breast/Chest wall only**	12 (31.6)	Ref	0.693						
**Breast/chest wall and nodes**	26 (68.4)	0.831(0.332-2.080)							
**Use of SIB or GTV boost**									
**No**	22 (57.9)	Ref	0.047	Ref	0.007	Ref	0.020	Ref	0.017
**SIB/GTV boost**	16 (42.1)	0.415(0.175-0.988)		0.003(0.000-0.214)		0.351(0.145-0.848)		0.363(0.146-0.837)	
**Cumulative dose to GTV (Gy)**									
**Median (range)**	50 (30-62.5)	0.988(0.939-1.039)	0.630						
**Cumulative dose to PTV (Gy)**									
**Median (range)**	45.5 (30-57.5)	1.015(0.955-1.078)	0.633						
**Cumulative BED to GTV (Gy), α/β=4**									
**Median (range)**	78.9 (51.5-111.3)	0.992(0.962-1.023)	0.613						
**Cumulative BED to PTV (Gy), α/β=4**									

**Median (range)**	75.0 (45.0-98.8)	1.011(0.975-1.048)	0.556		
**RT technique**					
**2D/3D**	8 (21.1)	Ref	0.190		
**IMRT/VMAT**	30 (78.9)	0.501(0.178-1.409)			
**RT compliance**					
**Complete the RT**	32 (84.2)	Ref	0.951		
**Incomplete the RT**	6 (15.8)	1.039(0.303-3.565)			
**Concurrent systemic therapy**					
**Yes**	21 (55.3)	0.537(0.226-1.275)	0.159		
**No**	17 (44.7)	Ref			
**Systemic therapy change at time of RT**					
**Yes**	4 (19.0)	4.743(1.163-19.351)	0.030		0.205
**No**	17 (81.0)	Ref			
**Symptom relief after RT (within 3 months after palliative RT)**					
**Yes**	31 (81.6)	0.203(0.078-0.527)	0.001	60.677(1.496-2461.623)	0.030
**No**	7 (18.4)	Ref		Ref	
**Surgery after RT**					
**Yes**	4 (10.5)	1.611(0.536-4.840)	0.396		
**No**	34 (89.5)	Ref			

OS, overall survival; HR, hazard ratio; CI, confidence interval; ECOG, Eastern Cooperative Oncology Group; ER, estrogen receptor; HER2, human epidermal growth factor receptor 2; RT, radiotherapy; SIB, simultaneous integrated boost; GTV, gross tumor volume; PTV, planning target volume; BED, biologically effective dose; IMRT, intensity-modulated radiation therapy; VMAT, volumetric modulated arc therapy; Ref, reference; NE, not estimable.

†Exploratory multivariable model including covariates significant in univariate analysis
‡ Parsimonious multivariable model including ≥3 prior lines of systemic therapy and SIB/GTV boost (25 OS events; EPV ≈12.5)

§Firth-penalized Cox proportional hazards regression performed as a sensitivity analysis to address potential small-sample bias

Supplementary Table S2. univariate and multivariate analyses for Local control

Variable	N(%)	Unadjusted HR (95% CI)	p-value	Adjusted HR (95% CI)	p-value
**Age (y)**					
**Median (range)**	56.5 (29-79)	0.982(0.884-1.091)	0.982		
**ECOG status**					
**0-1**	31 (81.6)	Ref	0.085		
**>2**	7 (18.4)	4.875(0.804-29.546)			
**Time from initial diagnosis (month)**					
**Median (range)**	14.3 (0.4-78.2)	0.986(0.938-1.036)	0.571		
**Receptor status**					
**Others**	25 (65.8)	Ref	0.073		
**ER-/HER2-**	13 (34.2)	4.468(0.870-22.946)			
**Histologic grade**					
**Others**	27 (71.1)	Ref	0.299		
**High**	11 (28.9)	0.015(0.000-40.604)			
**Lines of systemic therapy before RT**					
**<3**	31 (81.6)	Ref	0.053		
**≥3**	7 (18.4)	5.989(0.976-36.762)			
**Prior upfront surgery**					
**Yes**	6 (15.8)	1.227(0.209-7.196)	0.821		
**No**	32 (68.4)	Ref			
**Clinical T stage at time of RT**					
**Others**	12 (31.6)	Ref	0.848		
**T4**	26 (68.4)	0.847(0.154-4.650)			
**Clinical N stage at time of RT**					
**Others**	10 (26.3)	Ref	0.606		
**N3**	28 (73.7)	1.212(0.583-2.517)			
**Clinical M stage at time of RT**					
**M0**	3 (7.9)	Ref	0.625		
**M1**	35 (92.1)	1.775(0.177-17.754)			
**metastasis (≤5 sites)**	22 (62.9)	Ref	0.597		
**Systemic metastasis (>5 sites)**	13 (37.1)	0.553(0.062-4.970)			
**Number of presenting symptoms**					
**<3**	24 (63.2)	Ref	0.253		
**≥3**	14 (36.8)	2.609(0.504-13.496)			
**Radiation target volume**					
**Mass only, Breast/Chest wall only**	12 (31.6)	Ref	0.626		
**Breast/chest wall and nodes**	26 (68.4)	1.726(0.193-15.472)			
**Use of SIB or GTV boost**					
**No**	22 (57.9)	Ref	0.338		

**SIB/GTV boost**	16 (42.1)	2.251(0.429-11.830)			
**Cumulative dose to GTV (Gy)**					
**Median (range)**	50 (30-62.5)	0.942(0.855-1.038)	0.226		
**Cumulative dose to PTV (Gy)**					
**Median (range)**	45.5 (30-57.5)	0.872(0.772-0.984)	0.026		0.849
**Cumulative BED to GTV (Gy), α/β=4**					
**Median (range)**	78.9 (51.5-111.3)	0.978(0.918-1.042)	0.493		
**Cumulative BED to PTV (Gy), α/β=4**					
**Median (range)**	75.0 (45.0-98.8)	0.909(0.839-0.985)	0.019	0.909(0.839-0.985)	0.019
**RT technique**					
**2D/3D**	8 (21.1)	Ref	0.539		
**IMRT/VMAT**	30 (78.9)	26.523(0.001-NE)			
**RT compliance**					
**Complete the RT**	32 (84.2)	Ref	0.710		
**Incomplete the RT**	6 (15.8)	1.516(0.169-13.596)			
**Concurrent systemic therapy**					
**Yes**	21 (55.3)	0.649(0.121-3.467)	0.613		
**No**	17 (44.7)	Ref			
**Systemic therapy change at time of RT**					
**Yes**	4 (19.0)	5.508(0.341-88.894)	0.229		
**No**	17 (81.0)	Ref			
**Symptom relief after RT (within 3 months after palliative RT)**					
**Yes**	31 (81.6)	0.108(0.017-0.667)	0.017		0.138
**No**	7 (18.4)	Ref			
**Surgery after RT**					
**Yes**	4 (10.5)	0.043(0.000-NE)	0.638		
**No**	34 (89.5)	Ref			

HR, hazard ratio; CI, confidence interval; ECOG, Eastern Cooperative Oncology Group; ER, estrogen receptor; HER2, human epidermal growth factor receptor 2; RT, radiotherapy; SIB, simultaneous integrated boost; GTV, gross tumor volume; PTV, planning target volume; BED, biologically effective dose; IMRT, intensity-modulated radiation therapy; VMAT, volumetric modulated arc therapy; Ref, reference; NE, not estimable.

Supplementary Table S3. univariate and multivariate analyses for Progression free survival

Variable	N(%)	Unadjusted HR (95% CI)	p-value	Adjusted HR (95% CI)	p-value
**Age (y)**					
**Median (range)**	56.5 (29-79)	0.988(0.943-1.036)	0.624		
**ECOG status**					
**0-1**	31 (81.6)	Ref	0.127		
**>2**	7 (18.4)	1.986(0.823-4.791)			
**Time from initial diagnosis (month)**					
**Median (range)**	14.3 (0.4-78.2)	0.968(0.939-0.999)	0.041	0.871(0.764-0.992)	0.037
**Receptor status**					
**Others**	25 (65.8)	Ref	<0.001	42.483(3.271-551.716)	0.004
**ER-/HER2-**	13 (34.2)	1.410(1.156-1.719)			
**Histologic grade**					
**Others**	27 (71.1)	Ref	0.067		
**High**	11 (28.9)	2.044(0.952-4.388)			
**Lines of systemic therapy before RT**					
**<3**	31 (81.6)	Ref	0.149		
**≥3**	7 (18.4)	1.973(0.784-4.967)			
**Prior upfront surgery**					
**Yes**	6 (15.8)	1.582(0.639-3.918)	0.321		
**No**	32 (68.4)	Ref			
**Clinical T stage at time of RT**					
**Others**	12 (31.6)	Ref	0.738		
**T4**	26 (68.4)	0.870(0.384-1.970)			
**Clinical N stage at time of RT**					
**Others**	10 (26.3)	Ref	0.135		
**N3**	28 (73.7)	2.095(0.795-5.518)			
**Clinical M stage at time of RT**					
**M0**	3 (7.9)	Ref	0.593		
**M1**	35 (92.1)	1.399(0.408-4.798)			
**metastasis (≤5 sites)**	22 (62.9)	Ref	0.324		
**Systemic metastasis (>5 sites)**	13 (37.1)	1.486(0.677-3.260)			
**Number of presenting symptoms**					
**<3**	24 (63.2)	Ref	0.138		
**≥3**	14 (36.8)	1.778(0.831-3.807)			
**Radiation target volume**					
**Mass only, Breast/Chest wall only**	12 (31.6)	Ref	0.962		
**Breast/chest wall and nodes**	26 (68.4)	1.020(0.446-2.336)			
**Use of SIB or GTV boost**					
**No**	22 (57.9)	Ref	0.133		

**SIB/GTV boost**	16 (42.1)	0.540(0.242-1.206)			
**Cumulative dose to GTV (Gy)**					
**Median (range)**	50 (30-62.5)	0.996(0.949-1.046)	0.881		
**Cumulative dose to PTV (Gy)**					
**Median (range)**	45.5 (30-57.5)	1.012(0.953-1.075)	0.691		
**Cumulative BED to GTV (Gy), α/β=4**					
**Median (range)**	78.9 (51.5-111.3)	0.998(0.968-1.028)	0.893		
**Cumulative BED to PTV (Gy), α/β=4**					
**Median (range)**	75.0 (45.0-98.8)	1.011(0.973-1.050)	0.580		
**RT technique**					
**2D/3D**	8 (21.1)	Ref	0.960		
**IMRT/VMAT**	30 (78.9)	1.025(0.387-2.718)			
**RT compliance**					
**Complete the RT**	32 (84.2)	Ref	0.840		
**Incomplete the RT**	6 (15.8)	0.896(0.310-2.592)			
**Concurrent systemic therapy**					
**Yes**	21 (55.3)	0.345(0.161-0.739)	0.006		0.771
**No**	17 (44.7)	Ref			
**Systemic therapy change at time of RT**					
**Yes**	4 (19.0)	4.314(1.248-14.913)	0.021		0.407
**No**	17 (81.0)	Ref			
**Symptom relief after RT (within 3 months after palliative RT)**					
**Yes**	31 (81.6)	0.323(0.133-0.786)	0.013		0.085
**No**	7 (18.4)	Ref			
**Surgery after RT**					
**Yes**	4 (10.5)	0.756(0.222-2.574)	0.655		
**No**	34 (89.5)	Ref			

PFS, progression-free survival; HR, hazard ratio; CI, confidence interval; ECOG, Eastern Cooperative Oncology Group; ER, estrogen receptor; HER2, human epidermal growth factor receptor 2; RT, radiotherapy; SIB, simultaneous integrated boost; GTV, gross tumor volume; PTV, planning target volume; BED, biologically effective dose; IMRT, intensity-modulated radiation therapy; VMAT, volumetric modulated arc therapy.

Supplementary Table S4. univariate and multivariate analyses for Distant metastasis free survival

Variable	N(%)	Unadjusted HR (95% CI)	p-value	Adjusted HR (95% CI)	p-value
**Age (y)**					
**Median (range)**	56.5 (29-79)	0.990(0.943-1.041)	0.704		
**ECOG status**					
**0-1**	31 (81.6)	Ref	0.374		
**>2**	7 (18.4)	1.592(0.571-4.434)			
**Time from initial diagnosis (month)**					
**Median (range)**	14.3 (0.4-78.2)	0.980(0.951-1.007)	0.152		
**Receptor status**					
**Others**	25 (65.8)	Ref	<0.001	20.941(3.232-135.696)	0.001
**ER-/HER2-**	13 (34.2)	5.139(2.096-12.596)			
**Histologic grade**					
**Others**	27 (71.1)	Ref	0.089		
**High**	11 (28.9)	2.081(0.893-4.847)			
**Lines of systemic therapy before RT**					
**<2**	31 (81.6)	Ref	0.008		0.477
**≥2**	7 (18.4)	3.354(1.363-8.254)			
**Prior upfront surgery**					
**Yes**	6 (15.8)	2.017(0.792-5.139)	0.141		
**No**	32 (68.4)	Ref			
**Clinical T stage at time of RT**					
**Others**	12 (31.6)	Ref	0.595		
**T4**	26 (68.4)	0.787(0.326-1.902)			
**Clinical N stage at time of RT**					
**Others**	10 (26.3)	Ref	0.355		
**N3**	28 (73.7)	1.597(0.592-4.308)			
**Clinical M stage at time of RT**					
**M0**	3 (7.9)	Ref	0.898		
**M1**	35 (92.1)	1.086(0.310-3.803)			
**metastasis (≤5 sites)**	22 (62.9)	Ref	0.708		
**Systemic metastasis (>5 sites)**	13 (37.1)	1.194(0.472-3.017)			
**Number of presenting symptoms**					
**<3**	24 (63.2)	Ref	0.074		
**≥3**	14 (36.8)	2.108(0.930-4.778)			
**Radiation target volume**					
**Mass only, Breast/Chest wall only**	12 (31.6)	Ref	0.858		
**Breast/chest wall and nodes**	26 (68.4)	0.921(0.374-2.269)			
**Use of SIB or GTV boost**					
**No**	22 (57.9)	Ref	0.235		

**SIB/GTV boost**	16 (42.1)	0.588(0.245-1.412)			
**Cumulative dose to GTV (Gy)**					
**Median (range)**	50 (30-62.5)	1.016(0.962-1.073)	0.571		
**Cumulative dose to PTV (Gy)**					
**Median (range)**	45.5 (30-57.5)	1.035(0.969-1.106)	0.302		
**Cumulative BED to GTV (Gy), α/β=4**					
**Median (range)**	78.9 (51.5-111.3)	1.009(0.977-1.042)	0.591		
**Cumulative BED to PTV (Gy), α/β=4**					
**Median (range)**	75.0 (45.0-98.8)	1.023(0.983-1.065)	0.269		
**RT technique**					
**2D/3D**	8 (21.1)	Ref	0.524		
**IMRT/VMAT**	30 (78.9)	1.488(0.438-5.051)			
**RT compliance**					
**Complete the RT**	32 (84.2)	Ref	0.809		
**Incomplete the RT**	6 (15.8)	1.143(0.387-3.374)			
**Concurrent systemic therapy**					
**Yes**	21 (55.3)	0.425(0.188-0.961)	0.040		0.067
**No**	17 (44.7)	Ref			
**Systemic therapy change at time of RT**					
**Yes**	4 (19.0)	6.010(1.588-22.750)	0.008	7.191(1.304-39.638)	0.024
**No**	17 (81.0)	Ref			
**Symptom relief after RT (within 3 months after palliative RT)**					
**Yes**	31 (81.6)	0.293(0.112-0.766)	0.012		0.084
**No**	7 (18.4)	Ref			
**Surgery after RT**					
**Yes**	4 (10.5)	1.276(0.377-4.324)	0.695		
**No**	34 (89.5)	Ref			

DMFS, distant metastasis-free survival; HR, hazard ratio; CI, confidence interval; ECOG, Eastern Cooperative Oncology Group; ER, estrogen receptor; HER2, human epidermal growth factor receptor 2; RT, radiotherapy; SIB, simultaneous integrated boost; GTV, gross tumor volume; PTV, planning target volume; BED, biologically effective dose; IMRT, intensity-modulated radiation therapy; VMAT, volumetric modulated arc therapy.

Supplementary Table S5. Radiologic response rate (within 6 months after palliative RT)

Radiologic response	N=35	Prescribed RT regimen (N)
**Complete response**	2	50 Gy/25fx -> 10 Gy/5fx and (56/46 Gy)/20fx
**Partial response**	16	Others (6), 50 Gy/20fx (4), 45 Gy/15fx (3), (50/44 Gy)/20fx (2), 41.6 Gy/16fx -> boost 10 Gy/ 4fx (1)
**Stable disease**	15	others (6), 50 Gy/25fx (5), 41.6 Gy/16fx -> boost 10 Gy/ 4fx (3), (50/44 Gy)/20fx (1),
**Progressive disease**	1	(62.5/50 Gy)/20fx
**Mixed response**	1	57.5 Gy/25fx

RT, radiotherapy; fx, fractions.

Supplementary Table S6. Treatment-related toxicities

Acute toxicity	N(%)
**Grade 0**	26 (68.4)
**Grade 1**	2 (5.3)
**Grade 2**	7 (18.4)
**Grade 3**	3 (7.9)
**Grade≥4**	0 (0)
**Late toxicity**	**N(%)**
**Grade 0**	30 (78.9)
**Grade 1**	8 (21.1)
**Grade≥2**	0 (0)
