# Supplementary material for: Clinical Outcomes of Palliative Radiotherapy for Breast Lesions in Symptomatic Advanced Breast Cancer: A Decade of Experience at a Regional Tertiary Hospital
Source: Cancers (Basel). 2026 Feb 27;18(5):769. doi: 10.3390/cancers18050769 (PMC12984870; doi:10.3390/cancers18050769)
Supplement: Supplementary file 1 [file cancers-18-00769-s001.zip › Supplementary Table.docx]

Supplementary Table S1. univariate and multivariate analyses for Overall Survival

| Variable | N(%) | Unadjusted HR (95% CI) | p-value | Adjusted HR†  (95% CI) | p-value | Parsimonious HR‡ (95% CI) | p-value | Firth-penalized HR§ (95% CI) | p-value |
| --- | --- | --- | --- | --- | --- | --- | --- | --- | --- |
| Age (y) |  |  |  |  |  |  |  |  |  |
| Median (range) | 56.5 (29-79) | 0.983(0.937-1.031) | 0.485 |  |  |  |  |  |  |
| ECOG status |  |  |  |  |  |  |  |  |  |
| 0-1 | 31 (81.6) | Ref | 0.023 |  | 0.563 |  |  |  |  |
| >2 | 7 (18.4) | 3.027(1.165-7.867) |  |  |  |  |  |  |  |
| Time from initial diagnosis (month) |  |  |  |  |  |  |  |  |  |
| Median (range) | 14.3 (0.4-78.2) | 0.973(0.940-1.008) | 0.126 |  |  |  |  |  |  |
| Receptor status |  |  |  |  |  |  |  |  |  |
| Others | 25 (65.8) | Ref | 0.001 |  | 0.587 |  |  |  |  |
| ER-/HER2- | 13 (34.2) | 3.954(1.729-9.045) |  |  |  |  |  |  |  |
| Histologic grade |  |  |  |  |  |  |  |  |  |
| Others | 27 (71.1) | Ref | 0.849 |  |  |  |  |  |  |
| High | 11 (28.9) | 1.094(0.434-2.757) |  |  |  |  |  |  |  |
| Lines of systemic therapy before RT |  |  |  |  |  |  |  |  |  |
| <3 | 31 (81.6) | Ref | 0.046 | Ref | 0.013 | Ref | 0.015 | Ref | 0.017 |
| ≥3 | 7 (18.4) | 2.687(1.020-7.082) |  | 42.081(2.217-798.745) |  | 3.500(1.278-9.590) |  | 3.632(1.285-9.387) |  |
| Prior upfront surgery |  |  |  |  |  |  |  |  |  |
| Yes | 6 (15.8) | 0.802(0.287-2.246) | 0.675 |  |  |  |  |  |  |
| No | 32 (68.4) | Ref |  |  |  |  |  |  |  |
| Clinical T stage at time of RT |  |  |  |  |  |  |  |  |  |
| Others | 12 (31.6) | Ref | 0.589 |  |  |  |  |  |  |
| T4 | 26 (68.4) | 0.787(0.331-1.876) |  |  |  |  |  |  |  |
| Clinical N stage at time of RT |  |  |  |  |  |  |  |  |  |
| Others | 10 (26.3) | Ref | 0.250 |  |  |  |  |  |  |
| N3 | 28 (73.7) | 2.061(0.601-7.072) |  |  |  |  |  |  |  |
| Clinical M stage at time of RT |  |  |  |  |  |  |  |  |  |
| M0 | 3 (7.9) | Ref | 0.302 |  |  |  |  |  |  |
| M1 | 35 (92.1) | 2.191(0.494-9.719) |  |  |  |  |  |  |  |
| metastasis (≤5 sites) | 22 (62.9) | Ref | 0.141 |  |  |  |  |  |  |
| Systemic metastasis (>5 sites) | 13 (37.1) | 1.913(0.806-4.537) |  |  |  |  |  |  |  |
| Number of presenting symptoms |  |  |  |  |  |  |  |  |  |
| <3 | 24 (63.2) | Ref | 0.040 | Ref | 0.011 |  |  |  |  |
| ≥3 | 14 (36.8) | 2.332(1.038-5.240) |  | 32.705(2.256-474.092) |  |  |  |  |  |
| Radiation target volume |  |  |  |  |  |  |  |  |  |
| Mass only, Breast/Chest wall only | 12 (31.6) | Ref | 0.693 |  |  |  |  |  |  |
| Breast/chest wall and nodes | 26 (68.4) | 0.831(0.332-2.080) |  |  |  |  |  |  |  |
| Use of SIB or GTV boost |  |  |  |  |  |  |  |  |  |
| No | 22 (57.9) | Ref | 0.047 | Ref | 0.007 | Ref | 0.020 | Ref | 0.017 |
| SIB/GTV boost | 16 (42.1) | 0.415(0.175-0.988) |  | 0.003(0.000-0.214) |  | 0.351(0.145-0.848) |  | 0.363(0.146-0.837) |  |
| Cumulative dose to GTV (Gy) |  |  |  |  |  |  |  |  |  |
| Median (range) | 50 (30-62.5) | 0.988(0.939-1.039) | 0.630 |  |  |  |  |  |  |
| Cumulative dose to PTV (Gy) |  |  |  |  |  |  |  |  |  |
| Median (range) | 45.5 (30-57.5) | 1.015(0.955-1.078) | 0.633 |  |  |  |  |  |  |
| Cumulative BED to GTV (Gy), α/β=4 |  |  |  |  |  |  |  |  |  |
| Median (range) | 78.9 (51.5-111.3) | 0.992(0.962-1.023) | 0.613 |  |  |  |  |  |  |
| Cumulative BED to PTV (Gy), α/β=4 |  |  |  |  |  |  |  |  |  |
| Median (range) | 75.0 (45.0-98.8) | 1.011(0.975-1.048) | 0.556 |  |  |  |  |  |  |
| RT technique |  |  |  |  |  |  |  |  |  |
| 2D/3D | 8 (21.1) | Ref | 0.190 |  |  |  |  |  |  |
| IMRT/VMAT | 30 (78.9) | 0.501(0.178-1.409) |  |  |  |  |  |  |  |
| RT compliance |  |  |  |  |  |  |  |  |  |
| Complete the RT | 32 (84.2) | Ref | 0.951 |  |  |  |  |  |  |
| Incomplete the RT | 6 (15.8) | 1.039(0.303-3.565) |  |  |  |  |  |  |  |
| Concurrent systemic therapy |  |  |  |  |  |  |  |  |  |
| Yes | 21 (55.3) | 0.537(0.226-1.275) | 0.159 |  |  |  |  |  |  |
| No | 17 (44.7) | Ref |  |  |  |  |  |  |  |
| Systemic therapy change at time of RT |  |  |  |  |  |  |  |  |  |
| Yes | 4 (19.0) | 4.743(1.163-19.351) | 0.030 |  | 0.205 |  |  |  |  |
| No | 17 (81.0) | Ref |  |  |  |  |  |  |  |
| Symptom relief after RT (within 3 months after palliative RT) |  |  |  |  |  |  |  |  |  |
| Yes | 31 (81.6) | 0.203(0.078-0.527) | 0.001 | 60.677(1.496-2461.623) | 0.030 |  |  |  |  |
| No | 7 (18.4) | Ref |  | Ref |  |  |  |  |  |
| Surgery after RT |  |  |  |  |  |  |  |  |  |
| Yes | 4 (10.5) | 1.611(0.536-4.840) | 0.396 |  |  |  |  |  |  |
| No | 34 (89.5) | Ref |  |  |  |  |  |  |  |

OS, overall survival; HR, hazard ratio; CI, confidence interval; ECOG, Eastern Cooperative Oncology Group; ER, estrogen receptor; HER2, human epidermal growth factor receptor 2; RT, radiotherapy; SIB, simultaneous integrated boost; GTV, gross tumor volume; PTV, planning target volume; BED, biologically effective dose; IMRT, intensity-modulated radiation therapy; VMAT, volumetric modulated arc therapy; Ref, reference; NE, not estimable.

†Exploratory multivariable model including covariates significant in univariate analysis
‡ Parsimonious multivariable model including ≥3 prior lines of systemic therapy and SIB/GTV boost (25 OS events; EPV ≈12.5)

§Firth-penalized Cox proportional hazards regression performed as a sensitivity analysis to address potential small-sample bias

Supplementary Table S2. univariate and multivariate analyses for Local control

| Variable | N(%) | Unadjusted HR (95% CI) | p-value | Adjusted HR (95% CI) | p-value |
| --- | --- | --- | --- | --- | --- |
| Age (y) |  |  |  |  |  |
| Median (range) | 56.5 (29-79) | 0.982(0.884-1.091) | 0.982 |  |  |
| ECOG status |  |  |  |  |  |
| 0-1 | 31 (81.6) | Ref | 0.085 |  |  |
| >2 | 7 (18.4) | 4.875(0.804-29.546) |  |  |  |
| Time from initial diagnosis (month) |  |  |  |  |  |
| Median (range) | 14.3 (0.4-78.2) | 0.986(0.938-1.036) | 0.571 |  |  |
| Receptor status |  |  |  |  |  |
| Others | 25 (65.8) | Ref | 0.073 |  |  |
| ER-/HER2- | 13 (34.2) | 4.468(0.870-22.946) |  |  |  |
| Histologic grade |  |  |  |  |  |
| Others | 27 (71.1) | Ref | 0.299 |  |  |
| High | 11 (28.9) | 0.015(0.000-40.604) |  |  |  |
| Lines of systemic therapy before RT |  |  |  |  |  |
| <3 | 31 (81.6) | Ref | 0.053 |  |  |
| ≥3 | 7 (18.4) | 5.989(0.976-36.762) |  |  |  |
| Prior upfront surgery |  |  |  |  |  |
| Yes | 6 (15.8) | 1.227(0.209-7.196) | 0.821 |  |  |
| No | 32 (68.4) | Ref |  |  |  |
| Clinical T stage at time of RT |  |  |  |  |  |
| Others | 12 (31.6) | Ref | 0.848 |  |  |
| T4 | 26 (68.4) | 0.847(0.154-4.650) |  |  |  |
| Clinical N stage at time of RT |  |  |  |  |  |
| Others | 10 (26.3) | Ref | 0.606 |  |  |
| N3 | 28 (73.7) | 1.212(0.583-2.517) |  |  |  |
| Clinical M stage at time of RT |  |  |  |  |  |
| M0 | 3 (7.9) | Ref | 0.625 |  |  |
| M1 | 35 (92.1) | 1.775(0.177-17.754) |  |  |  |
| metastasis (≤5 sites) | 22 (62.9) | Ref | 0.597 |  |  |
| Systemic metastasis (>5 sites) | 13 (37.1) | 0.553(0.062-4.970) |  |  |  |
| Number of presenting symptoms |  |  |  |  |  |
| <3 | 24 (63.2) | Ref | 0.253 |  |  |
| ≥3 | 14 (36.8) | 2.609(0.504-13.496) |  |  |  |
| Radiation target volume |  |  |  |  |  |
| Mass only, Breast/Chest wall only | 12 (31.6) | Ref | 0.626 |  |  |
| Breast/chest wall and nodes | 26 (68.4) | 1.726(0.193-15.472) |  |  |  |
| Use of SIB or GTV boost |  |  |  |  |  |
| No | 22 (57.9) | Ref | 0.338 |  |  |
| SIB/GTV boost | 16 (42.1) | 2.251(0.429-11.830) |  |  |  |
| Cumulative dose to GTV (Gy) |  |  |  |  |  |
| Median (range) | 50 (30-62.5) | 0.942(0.855-1.038) | 0.226 |  |  |
| Cumulative dose to PTV (Gy) |  |  |  |  |  |
| Median (range) | 45.5 (30-57.5) | 0.872(0.772-0.984) | 0.026 |  | 0.849 |
| Cumulative BED to GTV (Gy), α/β=4 |  |  |  |  |  |
| Median (range) | 78.9 (51.5-111.3) | 0.978(0.918-1.042) | 0.493 |  |  |
| Cumulative BED to PTV (Gy), α/β=4 |  |  |  |  |  |
| Median (range) | 75.0 (45.0-98.8) | 0.909(0.839-0.985) | 0.019 | 0.909(0.839-0.985) | 0.019 |
| RT technique |  |  |  |  |  |
| 2D/3D | 8 (21.1) | Ref | 0.539 |  |  |
| IMRT/VMAT | 30 (78.9) | 26.523(0.001-NE) |  |  |  |
| RT compliance |  |  |  |  |  |
| Complete the RT | 32 (84.2) | Ref | 0.710 |  |  |
| Incomplete the RT | 6 (15.8) | 1.516(0.169-13.596) |  |  |  |
| Concurrent systemic therapy |  |  |  |  |  |
| Yes | 21 (55.3) | 0.649(0.121-3.467) | 0.613 |  |  |
| No | 17 (44.7) | Ref |  |  |  |
| Systemic therapy change at time of RT |  |  |  |  |  |
| Yes | 4 (19.0) | 5.508(0.341-88.894) | 0.229 |  |  |
| No | 17 (81.0) | Ref |  |  |  |
| Symptom relief after RT (within 3 months after palliative RT) |  |  |  |  |  |
| Yes | 31 (81.6) | 0.108(0.017-0.667) | 0.017 |  | 0.138 |
| No | 7 (18.4) | Ref |  |  |  |
| Surgery after RT |  |  |  |  |  |
| Yes | 4 (10.5) | 0.043(0.000-NE) | 0.638 |  |  |
| No | 34 (89.5) | Ref |  |  |  |

HR, hazard ratio; CI, confidence interval; ECOG, Eastern Cooperative Oncology Group; ER, estrogen receptor; HER2, human epidermal growth factor receptor 2; RT, radiotherapy; SIB, simultaneous integrated boost; GTV, gross tumor volume; PTV, planning target volume; BED, biologically effective dose; IMRT, intensity-modulated radiation therapy; VMAT, volumetric modulated arc therapy; Ref, reference; NE, not estimable.

Supplementary Table S3. univariate and multivariate analyses for Progression free survival

| Variable | N(%) | Unadjusted HR (95% CI) | p-value | Adjusted HR (95% CI) | p-value |
| --- | --- | --- | --- | --- | --- |
| Age (y) |  |  |  |  |  |
| Median (range) | 56.5 (29-79) | 0.988(0.943-1.036) | 0.624 |  |  |
| ECOG status |  |  |  |  |  |
| 0-1 | 31 (81.6) | Ref | 0.127 |  |  |
| >2 | 7 (18.4) | 1.986(0.823-4.791) |  |  |  |
| Time from initial diagnosis (month) |  |  |  |  |  |
| Median (range) | 14.3 (0.4-78.2) | 0.968(0.939-0.999) | 0.041 | 0.871(0.764-0.992) | 0.037 |
| Receptor status |  |  |  |  |  |
| Others | 25 (65.8) | Ref | <0.001 | 42.483(3.271-551.716) | 0.004 |
| ER-/HER2- | 13 (34.2) | 1.410(1.156-1.719) |  |  |  |
| Histologic grade |  |  |  |  |  |
| Others | 27 (71.1) | Ref | 0.067 |  |  |
| High | 11 (28.9) | 2.044(0.952-4.388) |  |  |  |
| Lines of systemic therapy before RT |  |  |  |  |  |
| <3 | 31 (81.6) | Ref | 0.149 |  |  |
| ≥3 | 7 (18.4) | 1.973(0.784-4.967) |  |  |  |
| Prior upfront surgery |  |  |  |  |  |
| Yes | 6 (15.8) | 1.582(0.639-3.918) | 0.321 |  |  |
| No | 32 (68.4) | Ref |  |  |  |
| Clinical T stage at time of RT |  |  |  |  |  |
| Others | 12 (31.6) | Ref | 0.738 |  |  |
| T4 | 26 (68.4) | 0.870(0.384-1.970) |  |  |  |
| Clinical N stage at time of RT |  |  |  |  |  |
| Others | 10 (26.3) | Ref | 0.135 |  |  |
| N3 | 28 (73.7) | 2.095(0.795-5.518) |  |  |  |
| Clinical M stage at time of RT |  |  |  |  |  |
| M0 | 3 (7.9) | Ref | 0.593 |  |  |
| M1 | 35 (92.1) | 1.399(0.408-4.798) |  |  |  |
| metastasis (≤5 sites) | 22 (62.9) | Ref | 0.324 |  |  |
| Systemic metastasis (>5 sites) | 13 (37.1) | 1.486(0.677-3.260) |  |  |  |
| Number of presenting symptoms |  |  |  |  |  |
| <3 | 24 (63.2) | Ref | 0.138 |  |  |
| ≥3 | 14 (36.8) | 1.778(0.831-3.807) |  |  |  |
| Radiation target volume |  |  |  |  |  |
| Mass only, Breast/Chest wall only | 12 (31.6) | Ref | 0.962 |  |  |
| Breast/chest wall and nodes | 26 (68.4) | 1.020(0.446-2.336) |  |  |  |
| Use of SIB or GTV boost |  |  |  |  |  |
| No | 22 (57.9) | Ref | 0.133 |  |  |
| SIB/GTV boost | 16 (42.1) | 0.540(0.242-1.206) |  |  |  |
| Cumulative dose to GTV (Gy) |  |  |  |  |  |
| Median (range) | 50 (30-62.5) | 0.996(0.949-1.046) | 0.881 |  |  |
| Cumulative dose to PTV (Gy) |  |  |  |  |  |
| Median (range) | 45.5 (30-57.5) | 1.012(0.953-1.075) | 0.691 |  |  |
| Cumulative BED to GTV (Gy), α/β=4 |  |  |  |  |  |
| Median (range) | 78.9 (51.5-111.3) | 0.998(0.968-1.028) | 0.893 |  |  |
| Cumulative BED to PTV (Gy), α/β=4 |  |  |  |  |  |
| Median (range) | 75.0 (45.0-98.8) | 1.011(0.973-1.050) | 0.580 |  |  |
| RT technique |  |  |  |  |  |
| 2D/3D | 8 (21.1) | Ref | 0.960 |  |  |
| IMRT/VMAT | 30 (78.9) | 1.025(0.387-2.718) |  |  |  |
| RT compliance |  |  |  |  |  |
| Complete the RT | 32 (84.2) | Ref | 0.840 |  |  |
| Incomplete the RT | 6 (15.8) | 0.896(0.310-2.592) |  |  |  |
| Concurrent systemic therapy |  |  |  |  |  |
| Yes | 21 (55.3) | 0.345(0.161-0.739) | 0.006 |  | 0.771 |
| No | 17 (44.7) | Ref |  |  |  |
| Systemic therapy change at time of RT |  |  |  |  |  |
| Yes | 4 (19.0) | 4.314(1.248-14.913) | 0.021 |  | 0.407 |
| No | 17 (81.0) | Ref |  |  |  |
| Symptom relief after RT (within 3 months after palliative RT) |  |  |  |  |  |
| Yes | 31 (81.6) | 0.323(0.133-0.786) | 0.013 |  | 0.085 |
| No | 7 (18.4) | Ref |  |  |  |
| Surgery after RT |  |  |  |  |  |
| Yes | 4 (10.5) | 0.756(0.222-2.574) | 0.655 |  |  |
| No | 34 (89.5) | Ref |  |  |  |

PFS, progression-free survival; HR, hazard ratio; CI, confidence interval; ECOG, Eastern Cooperative Oncology Group; ER, estrogen receptor; HER2, human epidermal growth factor receptor 2; RT, radiotherapy; SIB, simultaneous integrated boost; GTV, gross tumor volume; PTV, planning target volume; BED, biologically effective dose; IMRT, intensity-modulated radiation therapy; VMAT, volumetric modulated arc therapy.

Supplementary Table S4. univariate and multivariate analyses for Distant metastasis free survival

| Variable | N(%) | Unadjusted HR (95% CI) | p-value | Adjusted HR (95% CI) | p-value |
| --- | --- | --- | --- | --- | --- |
| Age (y) |  |  |  |  |  |
| Median (range) | 56.5 (29-79) | 0.990(0.943-1.041) | 0.704 |  |  |
| ECOG status |  |  |  |  |  |
| 0-1 | 31 (81.6) | Ref | 0.374 |  |  |
| >2 | 7 (18.4) | 1.592(0.571-4.434) |  |  |  |
| Time from initial diagnosis (month) |  |  |  |  |  |
| Median (range) | 14.3 (0.4-78.2) | 0.980(0.951-1.007) | 0.152 |  |  |
| Receptor status |  |  |  |  |  |
| Others | 25 (65.8) | Ref | <0.001 | 20.941(3.232-135.696) | 0.001 |
| ER-/HER2- | 13 (34.2) | 5.139(2.096-12.596) |  |  |  |
| Histologic grade |  |  |  |  |  |
| Others | 27 (71.1) | Ref | 0.089 |  |  |
| High | 11 (28.9) | 2.081(0.893-4.847) |  |  |  |
| Lines of systemic therapy before RT |  |  |  |  |  |
| <2 | 31 (81.6) | Ref | 0.008 |  | 0.477 |
| ≥2 | 7 (18.4) | 3.354(1.363-8.254) |  |  |  |
| Prior upfront surgery |  |  |  |  |  |
| Yes | 6 (15.8) | 2.017(0.792-5.139) | 0.141 |  |  |
| No | 32 (68.4) | Ref |  |  |  |
| Clinical T stage at time of RT |  |  |  |  |  |
| Others | 12 (31.6) | Ref | 0.595 |  |  |
| T4 | 26 (68.4) | 0.787(0.326-1.902) |  |  |  |
| Clinical N stage at time of RT |  |  |  |  |  |
| Others | 10 (26.3) | Ref | 0.355 |  |  |
| N3 | 28 (73.7) | 1.597(0.592-4.308) |  |  |  |
| Clinical M stage at time of RT |  |  |  |  |  |
| M0 | 3 (7.9) | Ref | 0.898 |  |  |
| M1 | 35 (92.1) | 1.086(0.310-3.803) |  |  |  |
| metastasis (≤5 sites) | 22 (62.9) | Ref | 0.708 |  |  |
| Systemic metastasis (>5 sites) | 13 (37.1) | 1.194(0.472-3.017) |  |  |  |
| Number of presenting symptoms |  |  |  |  |  |
| <3 | 24 (63.2) | Ref | 0.074 |  |  |
| ≥3 | 14 (36.8) | 2.108(0.930-4.778) |  |  |  |
| Radiation target volume |  |  |  |  |  |
| Mass only, Breast/Chest wall only | 12 (31.6) | Ref | 0.858 |  |  |
| Breast/chest wall and nodes | 26 (68.4) | 0.921(0.374-2.269) |  |  |  |
| Use of SIB or GTV boost |  |  |  |  |  |
| No | 22 (57.9) | Ref | 0.235 |  |  |
| SIB/GTV boost | 16 (42.1) | 0.588(0.245-1.412) |  |  |  |
| Cumulative dose to GTV (Gy) |  |  |  |  |  |
| Median (range) | 50 (30-62.5) | 1.016(0.962-1.073) | 0.571 |  |  |
| Cumulative dose to PTV (Gy) |  |  |  |  |  |
| Median (range) | 45.5 (30-57.5) | 1.035(0.969-1.106) | 0.302 |  |  |
| Cumulative BED to GTV (Gy), α/β=4 |  |  |  |  |  |
| Median (range) | 78.9 (51.5-111.3) | 1.009(0.977-1.042) | 0.591 |  |  |
| Cumulative BED to PTV (Gy), α/β=4 |  |  |  |  |  |
| Median (range) | 75.0 (45.0-98.8) | 1.023(0.983-1.065) | 0.269 |  |  |
| RT technique |  |  |  |  |  |
| 2D/3D | 8 (21.1) | Ref | 0.524 |  |  |
| IMRT/VMAT | 30 (78.9) | 1.488(0.438-5.051) |  |  |  |
| RT compliance |  |  |  |  |  |
| Complete the RT | 32 (84.2) | Ref | 0.809 |  |  |
| Incomplete the RT | 6 (15.8) | 1.143(0.387-3.374) |  |  |  |
| Concurrent systemic therapy |  |  |  |  |  |
| Yes | 21 (55.3) | 0.425(0.188-0.961) | 0.040 |  | 0.067 |
| No | 17 (44.7) | Ref |  |  |  |
| Systemic therapy change at time of RT |  |  |  |  |  |
| Yes | 4 (19.0) | 6.010(1.588-22.750) | 0.008 | 7.191(1.304-39.638) | 0.024 |
| No | 17 (81.0) | Ref |  |  |  |
| Symptom relief after RT (within 3 months after palliative RT) |  |  |  |  |  |
| Yes | 31 (81.6) | 0.293(0.112-0.766) | 0.012 |  | 0.084 |
| No | 7 (18.4) | Ref |  |  |  |
| Surgery after RT |  |  |  |  |  |
| Yes | 4 (10.5) | 1.276(0.377-4.324) | 0.695 |  |  |
| No | 34 (89.5) | Ref |  |  |  |

DMFS, distant metastasis-free survival; HR, hazard ratio; CI, confidence interval; ECOG, Eastern Cooperative Oncology Group; ER, estrogen receptor; HER2, human epidermal growth factor receptor 2; RT, radiotherapy; SIB, simultaneous integrated boost; GTV, gross tumor volume; PTV, planning target volume; BED, biologically effective dose; IMRT, intensity-modulated radiation therapy; VMAT, volumetric modulated arc therapy.

Supplementary Table S5. Radiologic response rate (within 6 months after palliative RT)

| Radiologic response | N=35 | Prescribed RT regimen (N) |
| --- | --- | --- |
| Complete response | 2 | 50 Gy/25fx -> 10 Gy/5fx and (56/46 Gy)/20fx |
| Partial response | 16 | Others (6), 50 Gy/20fx (4), 45 Gy/15fx (3), (50/44 Gy)/20fx (2), 41.6 Gy/16fx -> boost 10 Gy/ 4fx (1) |
| Stable disease | 15 | others (6), 50 Gy/25fx (5), 41.6 Gy/16fx -> boost 10 Gy/ 4fx (3), (50/44 Gy)/20fx (1), |
| Progressive disease | 1 | (62.5/50 Gy)/20fx |
| Mixed response | 1 | 57.5 Gy/25fx |

RT, radiotherapy; fx, fractions.

Supplementary Table S6. Treatment-related toxicities

| Acute toxicity | N(%) |
| --- | --- |
| Grade 0 | 26 (68.4) |
| Grade 1 | 2 (5.3) |
| Grade 2 | 7 (18.4) |
| Grade 3 | 3 (7.9) |
| Grade≥4 | 0 (0) |
| Late toxicity | **N(%)** |
| Grade 0 | 30 (78.9) |
| Grade 1 | 8 (21.1) |
| Grade≥2 | 0 (0) |
