# Supplementary figures and images for: Clinical Outcomes of Palliative Radiotherapy for Breast Lesions in Symptomatic Advanced Breast Cancer: A Decade of Experience at a Regional Tertiary Hospital
Source: Cancers (Basel). 2026 Feb 27;18(5):769. doi: 10.3390/cancers18050769 (PMC12984870; doi:10.3390/cancers18050769)

## Slide 1
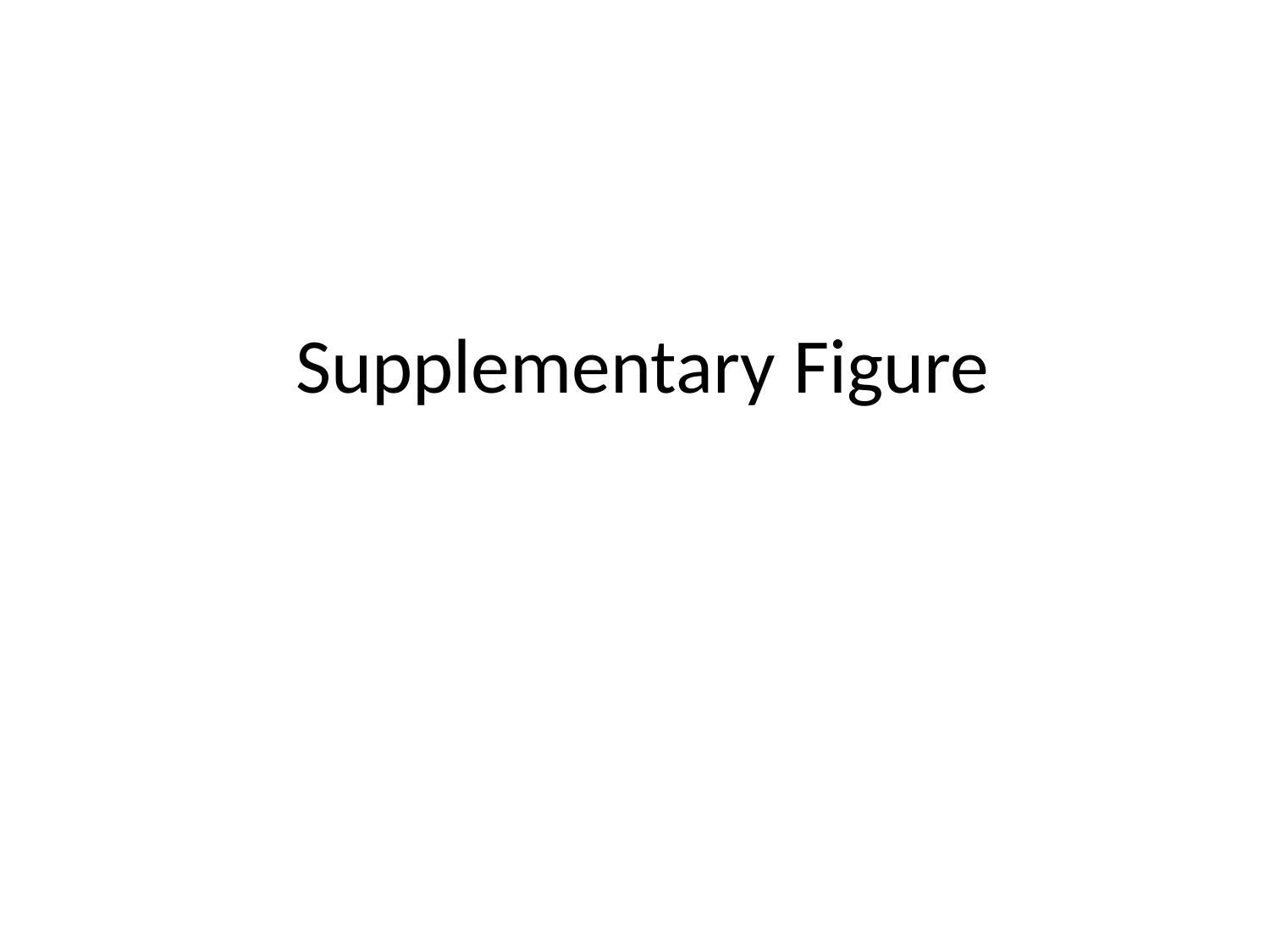

# Supplementary Figure

## Slide 2
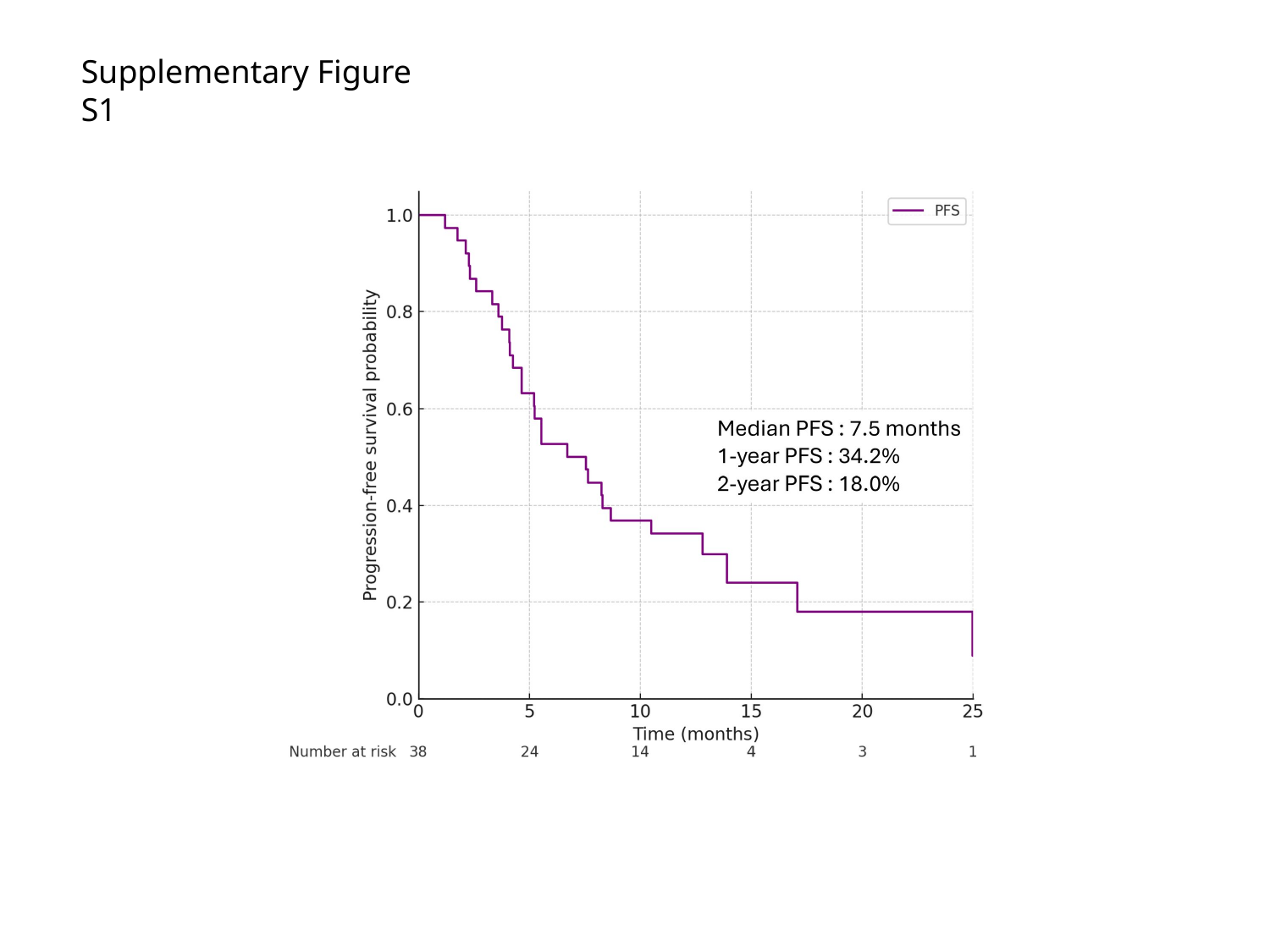

Supplementary Figure S1

## Slide 3
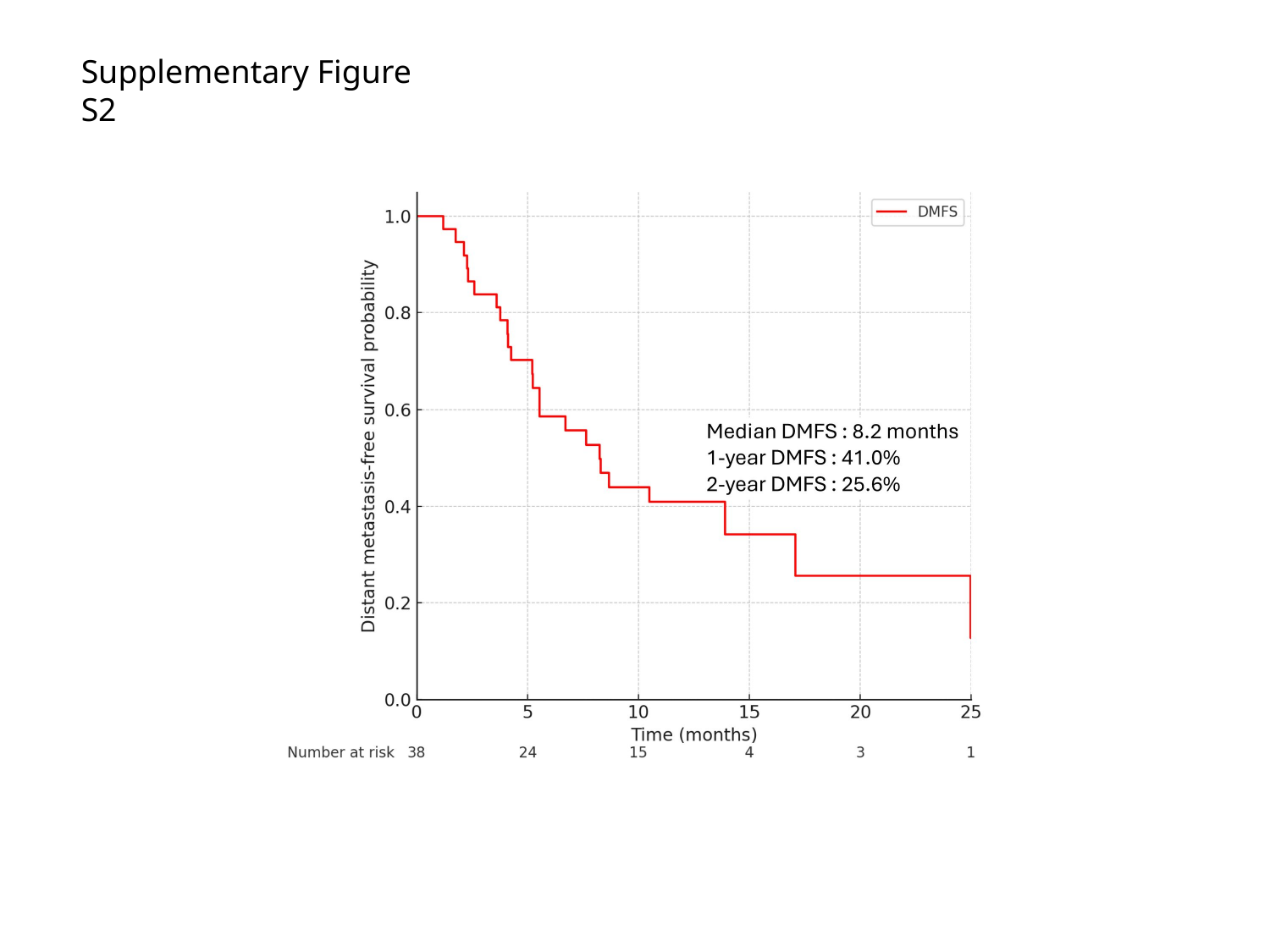

Supplementary Figure S2

Supplement: Supplementary file 1 [file cancers-18-00769-s001.zip › Supplementary Figure.pptx]
